# Epidural Analgesia With Surgical Stabilization of Flail Chest Following Blunt Thoracic Trauma in Patients With Multiple Trauma

**DOI:** 10.3389/fmed.2018.00280

**Published:** 2018-10-04

**Authors:** Darko Aleksa Golic, Dragan Svraka, Nataša Keleman, Snjezana Petrovic

**Affiliations:** Medical Faculty of Banja Luka, University Clinical Center of Republic of Srpska, University Banja Luka, Banjaluka, Bosnia and Herzegovina

**Keywords:** thoracic epidural analgesia, blunt thoracic trauma, multimodal analgesia, operative fixation of ribs, retrospective analysis

## Abstract

Flail chest, often defined as the fracture of three or more ribs in two or more places, represents the most severe form of rib fractures. Conservative treatment, consisting of respiratory assistance with endotracheal intubation and mechanical ventilation (internal pneumatic stabilization) and pain control, are the current treatments of choice in the majority of patients with multiple rib fractures. However, the use of mechanical ventilation may create complications. In selected patients, operative fixation of fractured ribs within 72 h post injury may lead to better outcomes. We conducted a retrospective analysis of a series of nine cases of patients who developed flail chest after blunt trauma, and were treated with surgical osteofixation of the chest wall and postoperative epidural analgesia at the University Clinical Center of the Republic of Srpska during the period from January 2015. to December 2016. Two patients had trauma to the chest only, and the other patients had associated injuries to the head, abdomen, spine, and fractures of the pelvis and long bones. In the majority of patients (77.7%), surgical stabilization of the chest was performed on the second day following the injury, (mean, 2.33 days) and no later than 5 days after the injury. All patients received epidural analgesia with 0, 25% bupivacaine and 0, 01% morphine and intravenous multimodal analgesia, beginning 6 h after thoracotomy. The average length of ICU stay was 14.7 days (range 2–36), while the average number of days of mechanical ventilation was 8.1. The average duration of hospitalization was 25.4 days. Tracheotomy was performed in 33.3% of study patients. Mortality in the observed group was 44.4%. This study shows that surgical stabilization and epidural analgesia reduced ventilator support, shortened trauma intensive care unit stay, and reduced medical costs vs internal pneumatic stabilization.

## Introduction

Traumatic flail chest is a potentially life threatening injury, often associated with prolonged mechanical ventilation and intensive care unit stay. Unfortunately, there is little literature describing the incidence of admission to the ICU following blunt trauma. In one small series of patients, Veysi et al. found that 37.5% of their blunt trauma population were admitted to the ICU and reported a mortality rate of 18.7%. Their mortality rate is similar to that in other published series (1). These patients often have multiple other injuries that complicate thoracic wall treatment options. Rib fractures occur in 10% of all trauma patients, and in approximately 30% of all patients with significant chest trauma, and are cause of significant morbidity (2, 3). Flail chest, generally defined as the fracture of three or more ribs in two or more places, represents the most severe form of rib fractures. Conservative therapies, consisting of respiratory assistance and pain control, are the current treatments for the majority of patients with multiple rib fractures; however, mechanical ventilation >3 weeks is often associated with several ventilation related complications. In patient with flail chest who are not treated surgically, pneumonia develops in 27 to 70% of cases and carries a mortality rate of 51%. Morbidity and mortality increase with age and the number of ribs that are fractured. Patients older than 45 years and with more than four rib fractures are at significantly greater risk for poorer outcomes compared to the average patient (4). In selected patients, operative fixation of fractured ribs within 72 h post injury may lead to better results (5). More randomized control trials are needed to further determine who may benefit from surgical fixation of rib fractures. Intravenous analgesia is often use for pain relief, but is associated with significant adverse effects. Epidural analgesia offers the advantage of superior analgesia with the absence of the adverse effects of parenteral narcotic.

Despite many reports of blunt chest trauma management, the impact of chest injury severity on the outcome of patients with multiple trauma has been rarely described. Significant differences in mortality and morbidity have been recently reported between different health care institutions.

Epidural analgesia provides superior pain relief compared to other analgesic techniques and improves the pulmonary function tests of injured patients. Its use has been associated with an increase in tidal volume, functional residual capacity (FRC), lung compliance, vital capacity and PaO2 with reductions in airway resistance and the paradoxical movement of flail chest wall segments (6).

## Materials and methods

We conducted a retrospective analysis of nine cases of patients who developed flail chest following blunt trauma, and were treated with osteofixation of the chest wall and postoperative epidural analgesia at the University Clinical Center of the Republic of Srpska (UCC RS) in Banjaluka from January 2015 to December 2016. All patients received a thoracic computed tomography (CT) scan upon admission to assess the severity of thoracic trauma. Osteofixation was performed under general endotracheal anesthesia with postoperative epidural analgesia. Thoracic epidural analgesia was located to a site corresponding to the level of the rib fracture as that technique has demonstrated superior analgesia in patients with blunt thoracic trauma (5). A solution of 0, 25 % bupivacaine with 0, 01% morphine was delivered by continuous infusion. The surgical osteofixation involved a posterolateral thoracotomy with pleural exploration in order to simultaneously treat lesions of the parenchymal lung and bronchus, control intrathoracic hemorrhage, and remove intrapleural clot.

## Results

The analysis included nine patients. All patients were male, aged 34–76 years, mean age 50 years. Four patients (44.4%) were motor vehicle accident victims, while five patients (55.5%) were injured at work. All patients underwent a posterolateral thoracotomy with osteofixation of the chest wall using an osteosynthesis plate. All patients had contusions of the lung parenchyma, and four patients had pulmonary parenchyma lacerations. Two patients had trauma to the chest only.

All patients had fractures of at least four ribs (range 4–8) and one patient had a bilateral flail chest. Seven patients had other major non–chest injuries: one had fractured mandible, 3 had intra-abdominal injuries (1 patient had a liver laceration and 2 patients had a splenic rupture), 1 patient had an L3 compression fracture, and one patient had fractures of the pelvis and lower extremities. All patients were transfused with a minimum of two units of packed RBCs (440 ml) and two units of fresh frozen plasma (360 ml). All the patients received epidural analgesia with 0,25% bupivacaine and 0,01% morphine at a rate 5–10 ml/ h-1 to keep pain score at less than 5 (on a visual analog pain scale of 0–10). Intravenous multimodal analgesia with additional non-narcotic analgesics were started within 6 h after thoracotomy. In the majority of patients (77.7%), surgical stabilization of the chest was performed on the second day of the injury, on average within 2.33 days of the injury, and no later than 5 days after the admission. The average duration of hospitalization was 25.4 days. The average length of stay of patients in the ICU was 14.7 days (range 2–36), while the average number of days on mechanical ventilation was 8.1 (range 2–16). Tracheostomy was performed in 33.3% of study patients. The overall mortality in the observed group was 44.4%.

## Discussion

Recently there has been renewed interest in surgical stabilization of ribs fracture in patients with flail chest (5). However, conservative treatment is still preferred in most surgical centers. Nishiumi et al. reported on the treatment of anterior flail chest with internal pneumatic stabilization in 42 patients. Continuous positive pressure ventilation was needed for 12.5 days and mechanical ventilation for 15.6 days (7). The goal of operative chest wall stabilization is to shorten the period of mechanical ventilation and thus reduce complications from its use.

Only three randomized controlled trials have compared operative vs. nonoperative treatment of multiple rib fractures (5). Leinicke et al., in a systematic review and meta-analysis of studies totaling 538 patients with flail chest, showed that operative management of flail chest was associated with a shorter duration of mechanical ventilation [pooled reduction: −4.52 days; 95% confidence interval (CI): −5.54 to −3.50], shorter ICU length of stay (−3.40 days; 95% CI: −6.01 to −0.79), decreased hospital length of stay (−3.82 days; 95% CI: −7.12 to −0.54), and decreased mortality (pooled Relative Risk (RR): 0.44; 95% CI: 0.28–0.69). A reduction in pneumonia (RR:0.45; 95% CI: 0.30–0.69), and tracheostomy (RR:0.25; 95% CI: 0.13–0.47) were also noted (8). In our study the average number of days on mechanical ventilation was 8.1 days, similar to that reported in the above meta-analysis. In our series the average patient age was 50 years (range 34–76 years), higher than the average reported in other studies. Several studies have described increased morbidity and mortality in elderly patients with traumatic rib fractures, and the increased death rate of our study may be explained by this. An age of 45 years or greater and more than four rib fractures is associated with significantly poorer patient outcome (8). Mortality in patients over 65 years with rib fractures compared with a younger population is 20.1 vs. 11.4% (9). In one retrospective study which analyzed data from the US National Trauma Data Bank, Kent et al. reported that 56 % of the mortality in patients greater than 65 years with thoracic trauma was due to rib fractures and no other injuries (4).

Contusion of the lung parenchyma was present in all patients in our case series and is an independent risk factor for the development of respiratory dysfunction, pneumonia, and acute respiratory distress syndrome. Pneumonia and prolonged respiratory dysfunction occurs in the 25–30% of patients with blunt trauma to the chest (4). Alveolar capillaries are injured, which results in an accumulation of blood and other fluids within lung tissue and interfere with gas exchange and leading to hypoxemia. The consequences of pulmonary contusion include ventilation/perfusion mismatching, increased arterio-venous shunting and loss of compliance of lung parenchyma. These physiological consequences occur within few hours after injury and usually resolve in 7 days. In one study of 139 patients with blunt thoracic trauma and pulmonary contusion, Novakon et al. reported a mortality rate of 17% (9).

All patients in our series received postoperative epidural analgesia via a thoracic epidural catheter. In addition to epidural analgesia, all patients received intravenous multimodal analgesia beginning 6 hours after chest wall ostefixation. Our goal was to achieve a pain score of 5 or less on a visual analog scale pain, which would provide good analgesia. Neuraxial blockade offers superior analgesia to systemically administered medications, although small supplementation of opioids is often required. In patients with one or two rib fractures, treatment with a systemic NSAID and modest amounts of systemic opioids often provides sufficient analgesia. However, such analgesic techniques are often not sufficient to provide satisfactory pain relief in patients with a greater number of fractured ribs who require physiotherapy or to allow effective coughing. Thoracic epidural analgesia with a catheter sited at the mean dermatomal level of the broken ribs provides superior analgesia when measured by subjective pain scale and objective respiratory parameters. Despite better pain relief, a recent meta-analysis which compared thoracic epidural analgesia to intravenous opioid therapy showed no statistically significant difference in mortality, duration of mechanical ventilation, or length of stay in the ICU and hospital (6). There is increasing evidence that multimodal analgesia combined with regional analgesia techniques other than thoracic neuraxial blockade reduces the severity of acute pain after thoracic surgery with yet unknown impact on the incidence and severity of chronic thoracic pain. As an alternative to epidural analgesia, continuous intercostal nerve blockade with local anesthetic may be used in a large number of patients. Paravertebral blockade may provide analgesia similar to thoracic epidural in patients with thoracic trauma and may have with fewer side effects.

The selection of patients and the timing of surgical intervention play an important role in the success of surgical osteofixation. Surgical stabilization of rib fractures is currently used in less than 1% of patients with multiple rib fractures (8). In our case series 77.7% of surgical stabilizations were done on the second day following the injury. Early vs. later intervention may improve outcomes as inflammation and callus formation increase between 3 and 5 days after the injury may complicate management. Most authors recommend early operative treatment within 72 h of injury. Although the combination of osteofixation and continuous thoracic epidural analgesia may reduce mortality, our mortality rate was higher than that of other studies. This is most likely due to the older patients we treated (mean age, 50 years; range 34–76 years) all of whom had significant lung parenchymal injury and numerous other non-thoracic injuries. Our study's significance is limited by the small number of subjects.

## Conclusion

Early surgical fixation of a flail chest followed by continuous thoracic epidural analgesia may reduce the duration of mechanical ventilation, its complications and shorten the length of ICU and hospital stay in patients with flail chest following blunt thoracic trauma. Good analgesia may help to improve a patient's respiratory mechanics and to avoid intubation of the trachea for ventilatory support in some patients. This may dramatically improve the course of recovery.

## Author contributions

DG: Main author, idea of writing the analysis, coordinated writing of the text and translation in English; DS: Analysis of litherature, writing of introduction, discussion, some part of translatin in english; SP: Correction of the text, correction of english version; NK: Statistical analysis, analysis of English.

### Conflict of interest statement

The authors declare that the research was conducted in the absence of any commercial or financial relationships that could be construed as a potential conflict of interest.
